# A New Potent Inhibitor against α-Glucosidase Based on an In Vitro Enzymatic Synthesis Approach

**DOI:** 10.3390/molecules29040878

**Published:** 2024-02-16

**Authors:** Huanyu Zhang, Xiance Che, Hongyan Jing, Yaowu Su, Wenqi Yang, Rubing Wang, Guoqi Zhang, Jie Meng, Wei Yuan, Juan Wang, Wenyuan Gao

**Affiliations:** 1 School of Pharmaceutical Science and Technology, Tianjin University, Tianjin 300072, China; zhanghychn@163.com (H.Z.); suyaowu@tju.edu.cn (Y.S.); 2021213044@tju.edu.cn (W.Y.); 16611895197@163.com (R.W.); z1667351973@163.com (G.Z.); 18768153215@163.com (J.M.); 13311286486@163.com (W.Y.); 2 Key Laboratory of Systems Bioengineering, Ministry of Education, Tianjin University, Tianjin 300072, China; 3 School of Traditional Chinese Materia Medica, Tianjin University of Traditional Chinese Medicine, Tianjin 301600, China; chexiance@163.com (X.C.); wmyyana@163.com (H.J.)

**Keywords:** in vitro enzymatic synthesis, type II diabetes mellitus, network pharmacology, molecular docking, molecular dynamics simulation, site-directed mutagenesis

## Abstract

Inhibiting the activity of intestinal α-glucosidase is considered an effective approach for treating type II diabetes mellitus (T2DM). In this study, we employed an in vitro enzymatic synthesis approach to synthesize four derivatives of natural products (NPs) for the discovery of therapeutic drugs for T2DM. Network pharmacology analysis revealed that the betulinic acid derivative P3 exerted its effects in the treatment of T2DM through multiple targets. Neuroactive ligand–receptor interaction and the calcium signaling pathway were identified as key signaling pathways involved in the therapeutic action of compound P3 in T2DM. The results of molecular docking, molecular dynamics (MD) simulations, and binding free energy calculations indicate that compound P3 exhibits a more stable binding interaction and lower binding energy (−41.237 kcal/mol) with α-glucosidase compared to acarbose. In addition, compound P3 demonstrates excellent characteristics in various pharmacokinetic prediction models. Therefore, P3 holds promise as a lead compound for the development of drugs for T2DM and warrants further exploration. Finally, we performed site-directed mutagenesis to achieve targeted synthesis of betulinic acid derivative. This work demonstrates a practical strategy of discovering novel anti-hyperglycemic drugs from derivatives of NPs synthesized through in vitro enzymatic synthesis technology, providing potential insights into compound P3 as a lead compound for anti-hyperglycemic drug development.

## 1. Introduction

Diabetes is an increasingly prevalent chronic disease worldwide, which can increase the risk of cardiovascular diseases and other illnesses, making it a significant global public health concern. According to a report by the World Health Organization (WHO) in 2023, approximately 422 million people worldwide are affected by diabetes, with 48% of diabetes-related deaths occurring before the age of 70. Over the past few decades, the number of diabetes cases and prevalence have been steadily increasing, and it is projected that the number of diabetes patients will rise to 552 million by 2030 [1]. Type II diabetes mellitus (T2DM) is the most common type of diabetes, accounting for 90% of all diabetes cases [2]. Recent studies have indicated that the etiology of type II (non-insulin-dependent) diabetes mellitus involves various factors, including impaired insulin secretion, reduced insulin activity, environmental factors, and genetic factors related to insulin resistance [3]. Diabetes is also associated with various complications, including retinopathy, diabetic neuropathy, diabetic nephropathy, stroke, coronary artery disease, peripheral artery disease, liver fibrosis, pulmonary fibrosis, various forms of cognitive dysfunction, depression, and erectile dysfunction [4,5,6]. These complications cause significant distress to patients. Among them, postprandial hyperglycemia is a major risk factor for cardiovascular diseases. Therefore, controlling postprandial hyperglycemia is an effective treatment approach for managing complications associated with T2DM.

Research has shown that inhibiting the activity of intestinal α-glucosidase is an effective means of controlling postprandial hyperglycemia [7,8]. Currently, acarbose is one of the most common α-glucosidase inhibitors used to effectively control blood glucose levels. However, long-term use of these medications may lead to serious side effects such as insomnia, headache, and constipation [9]. Therefore, it is necessary to continue searching for more effective α-glucosidase inhibitors.

Recent studies have shown that puerarin and betulinic acid exhibit excellent blood-glucose-lowering activities [10,11]. Combining pumpkin polysaccharides with puerarin in medication can synergistically improve T2DM [12]. Betulinic acid exhibits better inhibitory effects on α-glucosidase than the drug acarbose at very low concentrations. Furthermore, different substitutions at the C-3 and C-28 positions of betulinic acid may influence its inhibitory activity [11]. Therefore, modifying active natural products (NPs) may be an effective approach to obtain compounds with excellent blood glucose-lowering properties.

The glycosylation of NPs using UDP-Glycosyltransferases (UGTs) can greatly influence the physicochemical properties of the molecules. For example, adding two glucose molecules to protopanaxadiol (PPD) can generate the excellent anti-cancer compound ginsenoside Rg3 [13]. Ginsenoside Rg3, as a monomeric anticancer drug, has been widely used in clinical practice. Currently, multiple plant-derived UGTs have been discovered from licorice, ginseng, and sanqi, which can catalyze the synthesis of various triterpenoid saponins and flavonoid compounds. Compared to plant-derived UGTs, microbial-derived UGTs exhibit advantages such as high heterologous expression levels, broad substrate specificity, and high catalytic activity [14]. Bs-YjiC, as a microbial-derived UGTs, has been reported to exhibit excellent substrate promiscuity [15]. Therefore, we utilized the microbial-derived UGT Bs-YjiC to glycosylate puerarin and betulinic acid to obtain NPs derivatives with high hypoglycemic activity.

## 2. Results and Discussion

### 2.1. Enzymatic Synthesis of Puerarin Derivatives with Bs-YjiC

**Figure 1:**
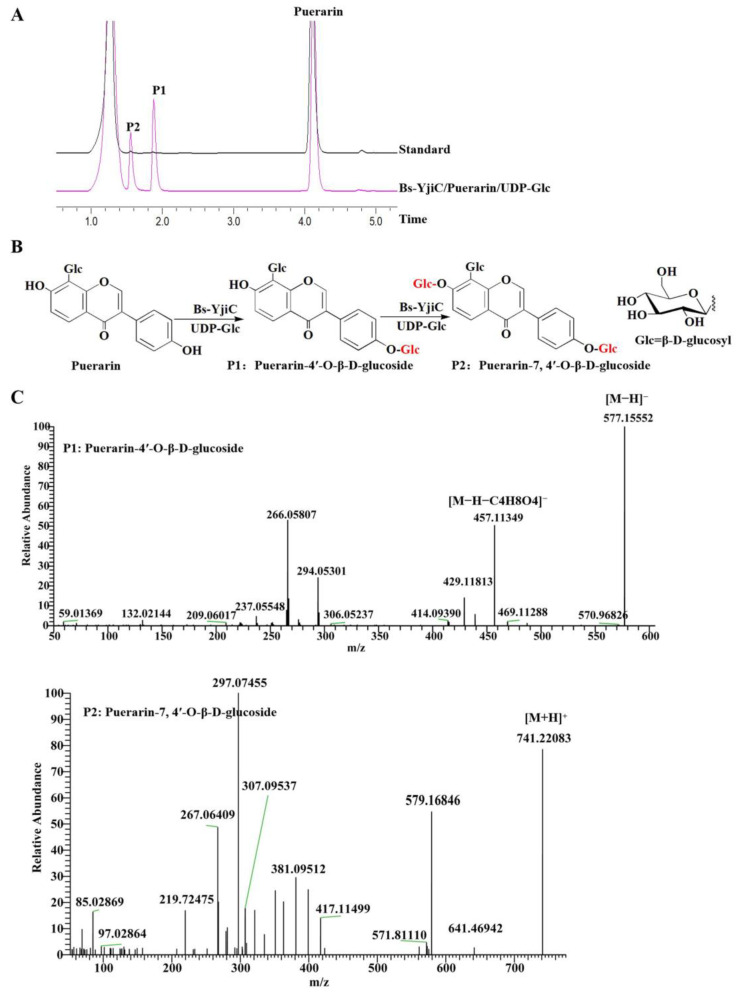
Analysis of the glycosylated products catalyzed by Bs-YjiC using puerarin as substrate. (**A**) HPLC analysis Bs-YjiC-catalyzed reactions. HPLC chromatograms were recorded at 254 nm; (**B**) glycosylation of substrate puerarin catalyzed by Bs-YjiC using UDP-Glc as a sugar donor; (**C**) LC-MS analysis of the glycosylated product catalyzed by Bs-YjiC.

We expressed the recombinant vector pET28a-Bs-YjiC in *Escherichia coli* BL21 (DE3) and conducted enzymatic activity verification to produce derivatives of puerarin. The results indicate that using puerarin as the substrate and UDP-Glc as the sugar donor, two products, P1 and P2, were synthesized under the catalysis of Bs-YjiC (Figure 1A). The liquid chromatography–mass spectrometry (LC-MS) results indicate that the molecular weight (Mw) of P1 is 577.1555, suggesting its molecular formula to be C_27_H_30_O_14_. Further analysis was conducted using tandem MS with the parent ion *m*/*z* 577.1555. The obtained tandem MS showed a prominent fragment ion at 457.1135 [M-H-C_4_H_8_O_4_]^−^, which is generated by the loss of a C_4_H_8_O_4_ (Mw 120) molecule from the parent molecular ion. The fragmentation pattern is consistent with that of puerarin-4′-*O*-β-d-glucoside. Therefore, P1 is identified as puerarin-4′-*O*-β-d-glucoside (Figure 1B). The molecular weight of P2 is 741.22083, suggesting its molecular formula to be C_33_H_40_O_19_, indicating the addition of two glucose units to puerarin. Based on the main function of Bs-YjiC in achieving the glycosylation of the mother ring-OH [16], it can be inferred that P2 is puerarin-7,4′-*O*-β-d-glucoside (Figure 1B). Therefore, we have successfully established a biosynthetic pathway for the glycosylation of puerarin.

### 2.2. Enzymatic Synthesis of Betulinic Acid Derivatives with Bs-YjiC

**Figure 2:**
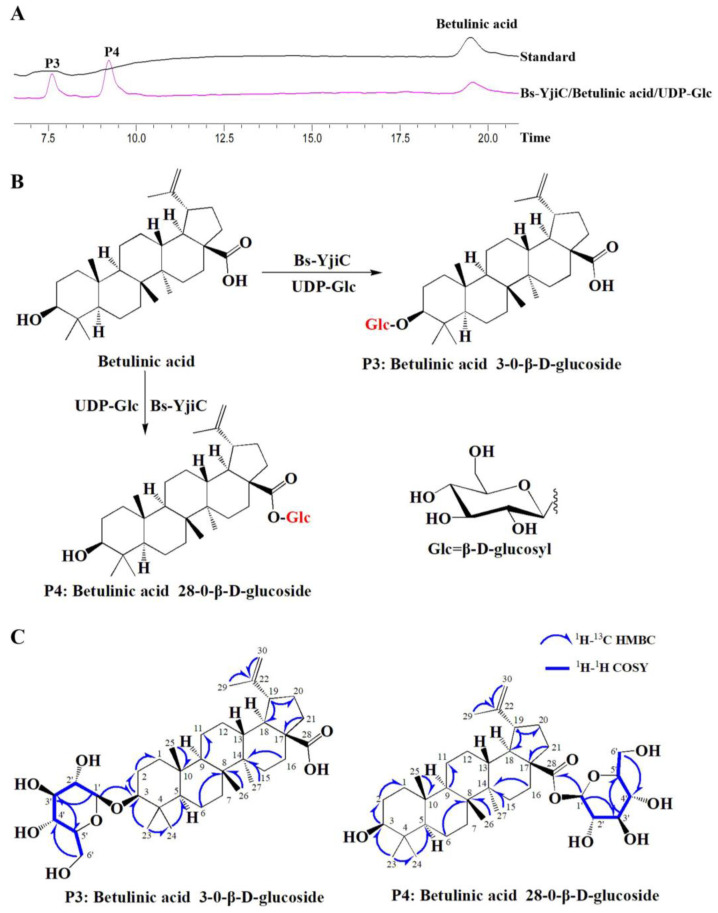
Analysis of the glycosylated products catalyzed by Bs-YjiC using betulinic acid as substrate. (**A**) HPLC analysis Bs-YjiC-catalyzed reactions. HPLC chromatograms were recorded at 210 nm; (**B**) glycosylation of substrate betulinic acid catalyzed by Bs-YjiC using UDP-Glc as a sugar donor; (**C**) key 2D NMR correlations of compound P3 and P4.

Using betulinic acid as a substrate and UDP-Glc as the sugar donor, the Bs-YjiC enzyme catalyzes the production of two compounds, P3 and P4 (Figure 2A). To analyze the structure of the product, P3, it was first purified using preparative HPLC. The structure of P3 was identified using 1D-NMR (^1^H-NMR and ^13^C-NMR) and 2D-NMR (HMBC, HSQC, and COSY) spectroscopy (Figures S1–S5). The ^1^H-NMR and ^13^C-NMR data indicated that the product P3 is a glycosylated derivative, with the addition of a glucose moiety to the substrate betulinic acid. By analyzing the ^1^H-^1^H COSY and HSQC correlations, we have assigned the pyranose glucose moiety (Table S1). The HSQC indicated that the signal for the terminal carbon of the sugar moiety is δC 106.7. The HMBC correlation between the anomeric proton signal H-1’ (δH 4.31, d, J = 7.8 Hz) and the C3 (δC 90.8) indicated that the glucose moiety is attached to the C3-OH of betulinic acid (Figure S4). Therefore, product P3 is identified as betulinic acid 3-*O*-β-d-glucopyranoside.

The product P4 was subsequently purified using preparative HPLC, and its structure was determined using 1D-NMR (^1^H-NMR and ^13^C-NMR) and 2D-NMR (HMBC, HSQC, and COSY) spectroscopy (Figures S6–S10). The ^1^H-NMR and ^13^C-NMR data revealed that P4 is a glycosylated derivative, with the addition of a glucose moiety to the substrate betulinic acid. By analyzing the ^1^H-^1^H COSY and HSQC correlations, we have assigned the pyranose glucose moiety (Table S2). The HSQC indicated that the signal for the terminal carbon of the sugar moiety is δC 95.3. The HMBC correlation between the anomeric proton signal H-1’ (δH 5.50, d, J = 6 Hz) and C28 (δC 176.9) indicated that the glucose moiety is attached to the C28-OH of betulinic acid (Figure S9). Therefore, the product P4 is identified as betulinic acid 28-*O*-β-d-glucopyranoside (Figure 2).

### 2.3. Common Targets Prediction

Using the SWISS target prediction database, the target proteins regulated by these four compounds were predicted, and after removing duplicates, a total of 38 catalytic target proteins were identified. The keywords “T2DM”, “Type II diabetes”, and “Type II diabetes mellitus” were used to screen disease-related targets in the GeneCards database. After merging and removing duplicates, a total of 17,727 targets associated with T2DM were obtained. A Venn diagram was generated by combining the disease targets and compound targets, resulting in 36 intersecting targets. These 36 targets are considered potential therapeutic targets for treating T2DM with these compounds (Figure S11).

### 2.4. Screening of PPI Networks and Core Targets

**Figure 3:**
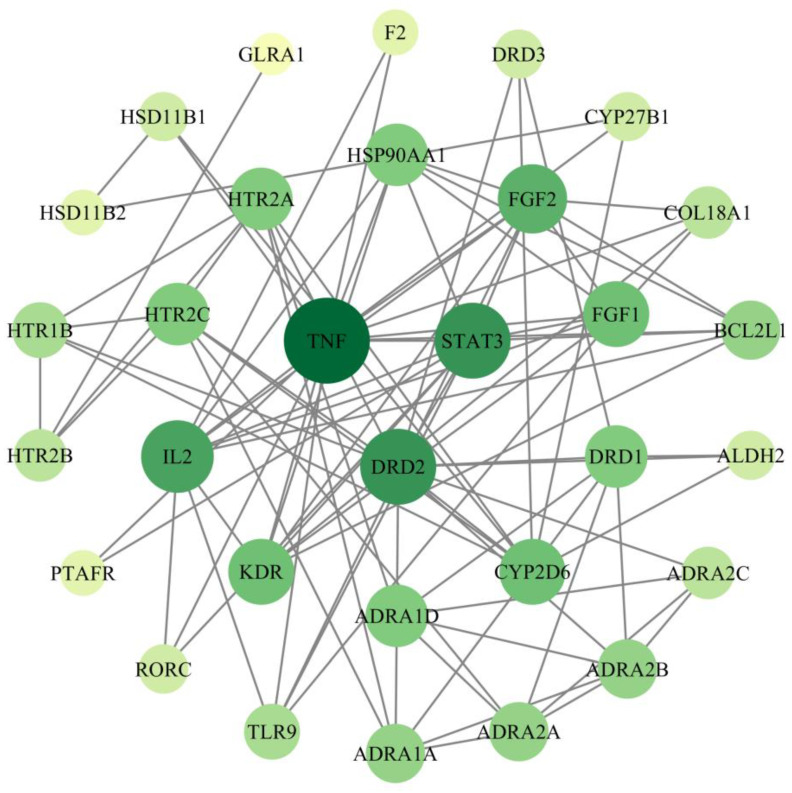
Screening of core targets.

A total of 36 intersection target points were imported into the STRING platform, with the species limited to “Homo sapiens” and the free target points hidden; a total of 31 core target points were obtained. The protein–protein interaction (PPI) data were downloaded and topological analysis was performed using Cytoscape 3.7.2. The network was sorted based on the degree centrality, and a PPI network resembling concentric circles was constructed. The network consists of 36 nodes and 91 edges, with an average node degree of 5.06 and an average local clustering coefficient of 0.55. The expected number of edges is 27, and the PPI enrichment has a *p*-value of <1.0 × 10^−16^. In the network, nodes represent proteins, and lines represent existing interactions. The darker and larger the color and size of a node, the higher its degree value, indicating that it interacts with more proteins and plays a more important role in the network. Among them, target points with a high degree and betweenness centrality are predicted as key targets for treating T2DM with compounds. According to Figure 3 and Table S3, the core target points mainly include TNF, DRD2, STAT3, IL2, FGF2, and FGF1.

### 2.5. GO Function and KEGG Pathway Enrichment Analysis

**Figure 4:**
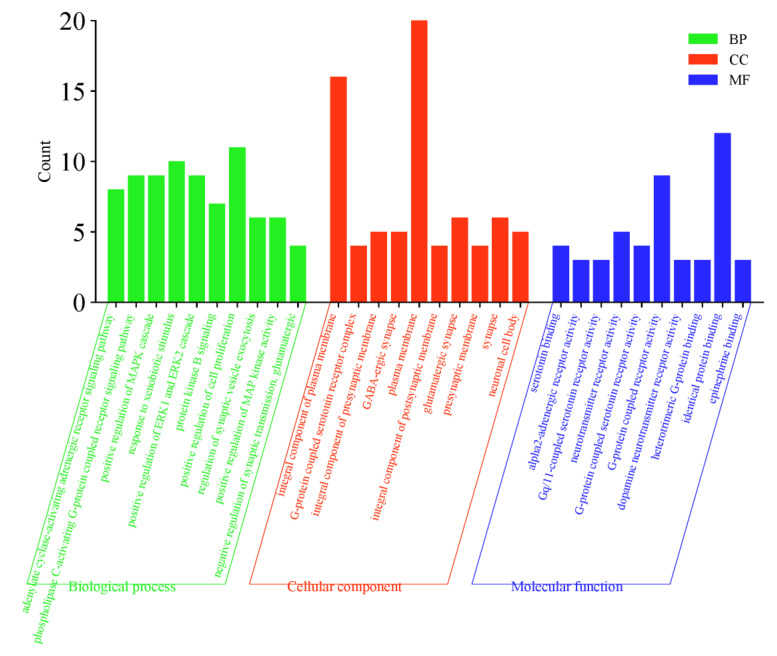
Core target GO analysis.

The 31 core target points were imported to the DAVID database to complete GO and KEGG analysis. GO analysis mainly includes three categories: biological process (BP), cellular component (CC), and molecular function (MF). There are 182 BPs involved, including adenylate cyclase-activating adrenergic receptor signaling pathway, positive regulation of MAPK cascade, response to xenobiotic stimulus, and other processes. A total of 26 CC entries were identified, including integral component of plasma membrane, G-protein coupled serotonin receptor complex, integral component of presynaptic membrane, and others. In addition, 27 MF items were found, including serotonin binding, alpha2-adrenergic receptor activity, gq/11-coupled serotonin receptor activity, and others (Figure 4).

**Figure 5:**
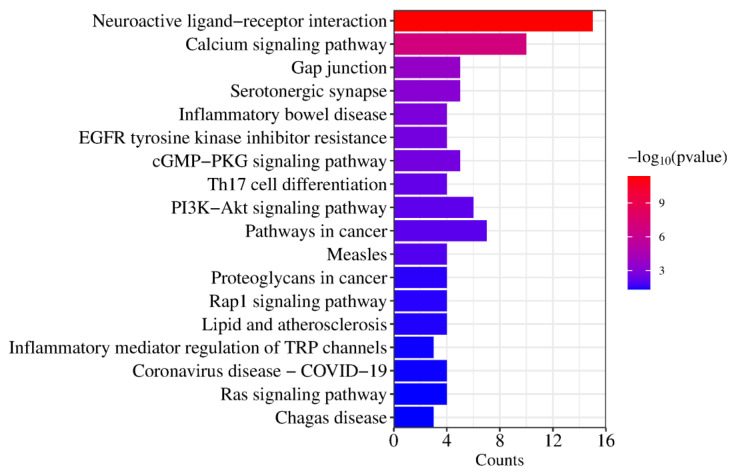
KEGG analysis of the core target.

KEGG pathway enrichment analysis revealed that the compounds exert their therapeutic effects on T2DM through 22 signaling pathways, primarily involving neuroactive ligand–receptor interaction, calcium signaling pathway, EGFR tyrosine kinase inhibitor resistance, PI3K-Akt signaling pathway, pathways in cancer, and other pathways (Figure 5 and Table 1).

### 2.6. The Drug–Target–Pathway–Disease Network

**Figure 6:**
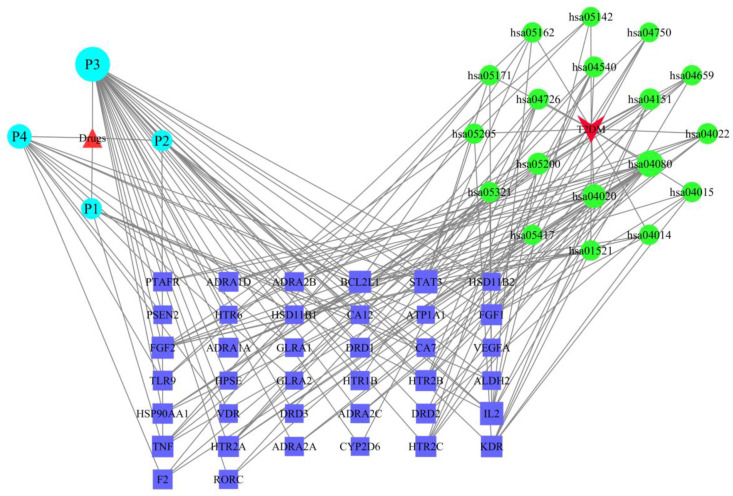
The drug–target–pathway–disease network.

Drugs, components, targets, diseases, and major KEGG enrichment pathways were imported into Cytoscape 3.7.2 to construct a network (Figure 6). In the network, nodes represent targets, drugs, components, pathways, or diseases, and edges represent interactions. In the graph, purple rectangles represent key targets, blue circles represent pathways, green circles represent components, upright triangles represent new compounds, and inverted triangles represent diseases. Topological analysis was performed on the network and the degree value of each node was calculated. The larger the node, the higher its degree value, indicating its greater importance in the network.

After analysis, it was found that compounds P3, P4, P2, and P1 have high degree values and closeness centrality, connecting to 30, 12, 6, and 6 targets, respectively. Among them, compound P3 has the highest degree value and closeness centrality, suggesting that compound P3 is likely the core compound for treating T2DM.

### 2.7. Molecular Docking and Molecular Dynamics Simulations of P3 with Core Targets

**Figure 7:**
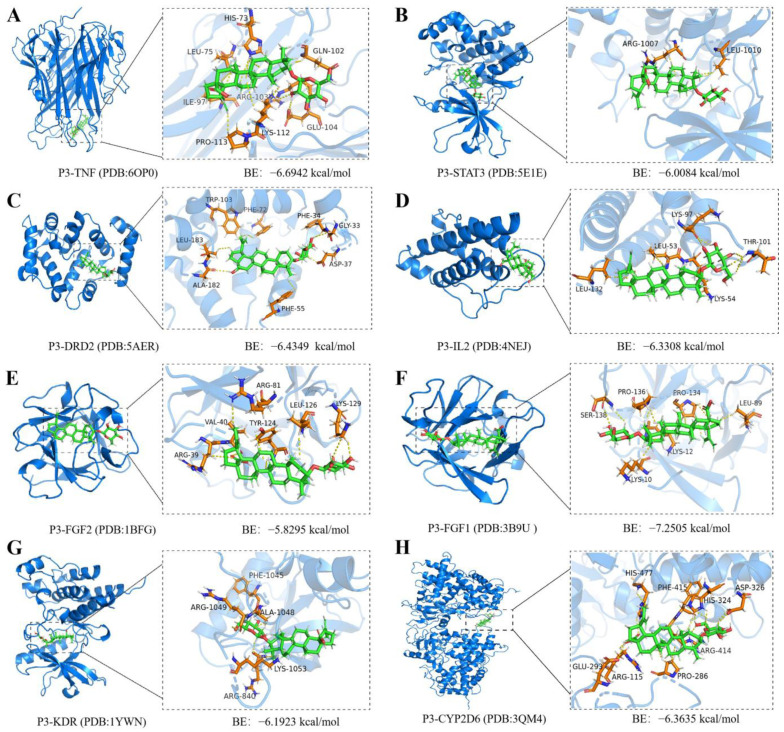
Molecular docking between compound P3 and key targets. (**A**) P3-TNF; (**B**) P3-STAT3; (**C**) P3-DRD2; (**D**) P3-IL2; (**E**) P3-FGF2; (**F**) P3-FGF1; (**G**) P3-KDR; (**H**) P3-CYP2D6.

Molecular docking methods can validate the binding interaction between small molecule drugs and potential targets, providing information for the potential therapeutic effects of small molecule drugs on diseases [17,18]. To investigate the interaction of compound P3 with different targets, we conducted molecular docking. According to the binding results, P3 can enter the binding domains of various target proteins and exhibits good binding affinity with all key targets (Figure 7). Therefore, P3 may exert its hypoglycemic effects by targeting these proteins.

The molecular docking energy range of P3 binding to different targets was −5.8295~−7.2505 kcal/mol. The lower the binding energy value obtained from molecular docking, the stronger the binding affinity between P3 and the protein target. Among them, the binding affinity between P3 and FGF1 is the strongest, with a docking energy of −7.2505 kcal/mol (Figure 7). The Ser138 residue on the FGF1 receptor forms a hydrogen bond interaction with P3. Residues Ser138 and Pro134 form van der Waals forces with P3, while residues Lys10, Pro136, Lys12, Leu89, and Pro134 on FGF1 form hydrophobic interaction forces with P3 (Figure S12).

Furthermore, we validated the binding ability between compound P3 and key target proteins through MD simulations. We selected the complex of compound P3 and FGF1 protein (PDB ID: 3B9U) for the MD simulations as they exhibited the strongest binding affinity during molecular docking. The equilibration of the simulation system was assessed using RMSD. As shown in Supplementary Figure S13A, the P3/FGF1 protein complex reached stability after 10 ns. The RMSF (Root Mean Square Fluctuation) in Supplementary Figure S13B reflects the flexibility of each residue in the molecule. Therefore, compound P3 may directly bind to the FGF1 protein to exert its pharmacological effects.

### 2.8. Molecular Docking and Molecular Dynamics Simulation of P3 and Acarbose with α-Glucosidase

The molecular docking method is one of methods for computer-aided drug design. Based on the known structure of ligand and target protein, small molecules are placed with three dimensional structures of known proteins. After optimizing the location of receptor compound, the best conformation of the interaction between the small molecules and the target macromolecules can be found. Molecular docking plays an important role in drug design and provides an effective tool for the discovery of leading compounds. Therefore, we conducted molecular docking of the synthesized product P3 with α-glucosidase to further screen for hypoglycemic lead compounds. Acarbose is a well-known anti-hyperglycemic drug that, due to its inhibitory effect on α-glucosidase, can be used to treat T2DM. Therefore, we also performed molecular docking using acarbose as the positive control drug and α-glucosidase as the target.

**Figure 8:**
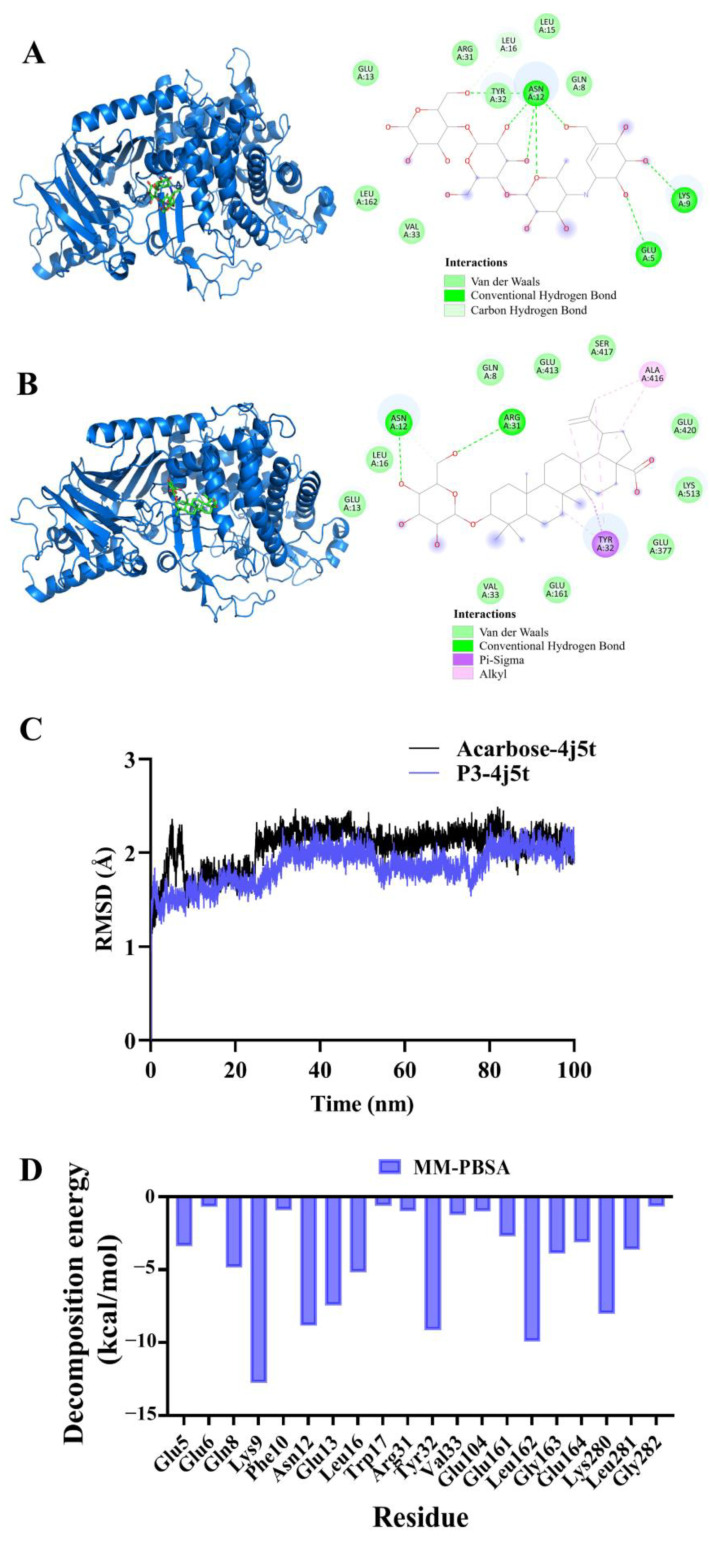
Molecular docking and molecular dynamics simulation of acarbose and P3 with α-glucosidase. (**A**) Molecular docking of acarbose with α-glucosidase; (**B**) molecular docking of P3 with α-glucosidase; (**C**) time dependence of root mean square deviation (RMSD) of the complex acarbose/α-glucosidase and P3/α-glucosidase; (**D**) decomposition of binding free energy of residues for α-glucosidase.

The binding interaction of P3 and acarbose to α-glucosidase was analyzed through molecular docking. As shown in Figure 8A, acarbose at the active pocket of α-glucosidase has high overlay, mainly due to Van de Walls interaction. Moreover, acarbose formed H-bonds to Glu A:5, Lys A:9, Asn A:12 of α-glucosidase. Asn A:12 is the most important residue, which formed five H-bonds with α-glucosidase to stabilize the orientation of the interaction. Compound P3 interacted with α-glucosidase more strongly. As shown in Figure 8B, compound 3 formed H-bonds to Asn A:12, Arg A:31 of α-glucosidase. Asn A:12 also formed the most H-bonds. Moreover, P3 demonstrated alkyl interaction with Tyr A:32, with Ala A:416 of α-glucosidase to lower the binding energy.

To further study the affinity between compound P3 and α-glucosidase, we performed 100 ns MD simulations (Figure 8C). As seen in Figure 8C, P3/α-glucosidase has relatively high stability compared to acarbose/α-glucosidase. The binding conformations of acarbose/α-glucosidase and P3/α-glucosidase are used to calculate the binding free energy using the MM/PBSA method. We found that the calculated binding free energies for acarbose and P3 in complexes acarbose/α-glucosidase and P3/α-glucosidase were −39.798 and −41.237 kcal/mol, respectively. The lower binding free energy of P3/α-glucosidase shows that P3 may be a better inhibitor of α-glucosidase than acarbose. To further elucidate the key amino acid residues that contribute more to the binding free energy, each residue decomposition is used to generate a residual–product interaction spectrum. As seen in Figure 8D, amino acid residues Lys9, Asn 12, Glu13, Tyr32, Leu162, and Lys280 interact strongly with P3 in the complex, suggesting that these amino acid residues may play an important role in the catalytic process.

### 2.9. Pharmacokinetic Prediction

ADMET analysis is an important tool in drug discovery [19,20]. We performed ADMET analysis on compound P3 using the ADMET-AI platform, and the results showed that compound P3 has excellent pharmacokinetic properties. As shown in Table 2, except for the plasma protein binding rate, the pharmacokinetic properties of the candidate drug P3 were not associated with any adverse effects in various prediction models, including human intestinal absorption, oral bioavailability, and blood–brain barrier permeability. Furthermore, compound P3 exhibited good biocompatibility in various toxicity prediction models, showing no evidence of clinical toxicity, mutagenicity, or carcinogenicity, which further suggests that compound P3 may be a promising lead compound for hypoglycemic agents.

### 2.10. Site-Directed Mutagenesis for the Directed Synthesis of Compound P3

Research has demonstrated that mutating the amino acid M315 of Bs-YjiC to phenylalanine leads to the generation of a mutant with enhanced C3 specificity towards protopanaxadiol, enabling the directed synthesis of ginsenoside Rh2 [21]. Therefore, to enhance the selectivity of the C3 position of betulinic acid and achieve the directed synthesis of compound P3, we performed site-directed mutagenesis on Bs-YjiC using the primers listed in Table S4. After a series of mutagenesis experiments, we obtained the mutant M315F. Subsequently, the mutant was purified, and its activity was verified. As shown in Figures S14 and S15, the M315F mutant showed more than 95% regional selectivity for the synthesis of compound P4 instead of P3, thus enabling directed synthesis of compound P4. Therefore, we can conclude that the mutant Bs-YjiC-M315F is highly site selective to the C28 position of betulinic acid.

## 3. Method

### 3.1. Materials and Reagents

Puerarin and betulinic acid were purchased from PUSH BIO-TECHNOLOGY (Chengdu, Sichuan, China). UDP-glucose (UDP-Glc) was purchased from RHAWN (Shanghai, China).

### 3.2. Heterogonous Expression and Purification of Bs-YjiC

Gene Bs-YjiC (NP_389104) was synthesized by the company GENEWIZ and inserted into the pET28a(+) expression vector, constructing the recombinant vector pET28a-Bs-YjiC. Then, the recombinant plasmid pET28a-Bs-YjiC was transformed into *Escherichia coli* DH5α. The recombinant *E. coli* DH5α strains were cultured in Luria-Bertani (LB) medium (50 mg/L kanamycin) at 37 °C, and the recombinant plasmid was extracted using a SPARKeasy Superpure Mini Plasmid Kit (Shandong Sparkjade Biotechnology Co., Ltd., Shandong, China). Then, the extracted recombinant plasmid was transformed into *Escherichia coli* BL21 (DE3) competent cells and the expression of the target protein was induced. The induction conditions for glycosyltransferase Bs-YjiC were as follows: it was cultivated in LB liquid medium containing 50 mg/L kanamycin (10 g/L NaCl, 10 g/L tryptone, and 5 g/L yeast extract) at 37 °C for approximately 4 h until the OD 600 of the culture reached 0.6–0.8. Then, isopropyl-β-d-thiogalactopyranoside (IPTG) was added to a final concentration of 0.5 mM and cultivation was continued at 18 °C and 180 r/min for 16 h to induce the expression of the target protein. Then, centrifugation was performed to collect the bacterial cells, which were then suspended in a 10 mM imidazole aqueous solution. Sonication was used to lyse the cells, and the protein-containing supernatant was loaded onto a Ni-NTA purification column for purification. The purified protein was eluted using a gradient of 40–400 mM imidazole.

### 3.3. Enzyme Activity Assay

In a 100 μL reaction system, including 0.5 mM puerarin and betulinic acid, 10 μg of glycosyltransferase Bs-YjiC, 5 mM UDP-Glucose, and 100 mM phosphate buffer (pH 7.5), the catalytic reaction was carried out at 37 °C for 12 h. This led to the formation of a mixture of puerarin and betulinic acid derivatives. The reaction was terminated by adding an equal volume of methanol. After centrifugation at 12,000 rpm for 10 min, the supernatant was collected and transferred to a liquid vial. The glycosylation products were then analyzed and identified using high-performance liquid chromatography (HPLC) and liquid chromatography–mass spectrometry (LC-MS). The chromatographic column used was a C18 column (4.6 mm × 250 mm, 5 μm particle) purchased from Kromasil, with an injection volume of 20 μL. A UV detector was used, and the flow rate of the mobile phase was set at 1 mL/min. The column temperature was maintained at 35 °C.

### 3.4. Structural Analysis of the Glycosylated Products

For the structural analysis of products P3 and P4, a scale-up reaction (500 mL) was prepared as described above. The reaction was terminated by adding an equal volume of methanol. Subsequently, the reaction mixture was evaporated to 5 mL. The products P3 and P4 were purified with a preparative HPLC system coupled with a reverse-phase C18 column (10 × 250 mm, 5 μm particles). After being evaporated, the purified products were re-dissolved in MeOH-*d*4. ^1^H-NMR, ^13^C-NMR, heteronuclear singular quantum correlation spectroscopy (HSQC), heteronuclear multiple-bond correlation spectroscopy (HMBC), and heteronuclear multiple-bond correlation spectroscopy (COSY) spectra were obtained using a 600 MHz NMR spectrometer.

### 3.5. Common Targets Prediction of Four Compounds and Type II Diabetes

The target proteins of four compounds were predicted using the online database SWISS target prediction (http://www.swisstargetprediction.ch/ (accessed on 3 December 2023)). Duplicates were organized, merged, and removed from the obtained target proteins to obtain the regulatory targets of the compounds. The keywords “T2DM”, “Type II diabetes”, and “Type II diabetes mellitus” were searched for in the GeneCards database (https://www.genecards.org/ (accessed on 3 December 2023)). The target proteins associated with T2DM were retrieved and organized. The obtained target proteins were merged and duplicates were removed to obtain the target proteins related to T2DM. Venny 2.1.0 was used to perform intersection analysis on the target proteins of compounds and T2DM, with the resulting intersection target proteins being potential therapeutic targets of compounds for treating T2DM.

### 3.6. Construction of the Protein–Protein Interaction Network and Screening of Key Targets

The intersection target proteins were imported into the STRING database (https://string-db.org/cgi/ (accessed on 3 December 2023)) for protein–protein interaction (PPI) analysis. The database was saved in TSV format and import into Cytoscape 3.7.2. The node diameter and color intensity were determined based on the node degree, and the core target proteins were filtered out.

### 3.7. GO Function and KEGG Pathway Enrichment Analysis of Key Targets

The core target genes for the treatment of T2DM by compounds were imported into the DAVID (https://david.ncifcrf.gov (accessed on 4 December 2023), v6.8) database. The species as set to “Homo sapiens” and gene ontology (GO) annotation and Kyoto Encyclopedia of Genes and Genomes (KEGG) pathway enrichment analysis were conducted. Then, the data were loaded onto a bioinformatics website for visualization.

### 3.8. Construction of the Drug–Target–Pathway–Disease Network

The nodes of disease, component, drug, core targets, and top 18 signaling pathways were imported into Cytoscape 3.7.2 to construct a drug–target–pathway–disease network, in order to determine the potential mechanism of compounds treatment for T2DM.

### 3.9. Molecular Docking, Molecular Dynamic Simulations and Calculation of Binding Free Energy

Using the crystal structure reported in the RCSB protein database (PDB: 7BOV) as a template, the homology modeling of Bs-YjiC was carried out using the Swiss Model platform [22]. The 100 ns MD simulation of P3/FGF1, acarbose/α-glucosidase and P3/α-glucosidase complexes was performed in Gromacs 2020. The topology files for acarbose and P3 were generated using the CHARMM 36 force field [23]. After the box is defined, the water solvation system is constructed using TIP3P water model [24]. The fastest descent method is used to optimize the potential energy of the system. After energy minimization and temperature equilibrium, the system gradually heats up in NVT (constant volume constant temperature) and then in NPT (constant pressure constant temperature). Finally, a 100 ns MD simulation was performed with a 2 fs timestep. We calculated the root mean square deviation (RMSD) using g_rms tools of GROMACS 2020. The MM/PBSA calculations were carried out using the g_mmpbsa package in GROMACS to calculate the binding free energy of acarbose/α-glucosidase and P3/α-glucosidase complexes [25].

### 3.10. Pharmacokinetic Prediction

The drug-like properties were predicted using the ADMET-AI online server (https://admet.ai.greenstonebio.com/ (accessed on 3 February 2024)). Compound P3 and acarbose were input into the ADMET-AI website and the ADMET properties were analyzed.

### 3.11. Site-Directed Mutagenesis

We performed site-directed mutagenesis using the wild-type Bs-YjiC as a template. The mutation primers are listed in Table S4. The mutants were created in the recombinant vector pET-28a-Bs-YjiC. PCR was performed using the Gloria Nova HS 2 × Master Mix (RK20717). Subsequently, the PCR products were subjected to seamless cloning using the 2 × MultiF Seamless Assembly Mix (RK21020) and transformed into Escherichia coli BL21(DE3) cells. Targeted mutations were confirmed by sequencing. After inducing recombinant *E. coli* expression, soluble enzyme fractions were obtained through cell sonication. SDS-PAGE was performed to test the protein purity after purification.

## 4. Conclusions

In vitro enzymatic catalysis is an effective approach for synthesizing active derivatives of NPs. In this study, four derivatives of NPs were synthesized using in vitro enzymatic catalysis, including puerarin-4′-*O*-β-d-glucoside (P1), puerarin-7,4′-*O*-β-d-glucoside (P2), betulinic acid 3-*O*-β-d-glucopyranoside (P3), and betulinic acid 28-*O*-β-d-glucopyranoside (P4). Subsequently, the hypoglycemic function of the synthesized derivatives of NPs was screened, and their hypoglycemic mechanisms were explored.

Network pharmacology studies have demonstrated that the betulinic acid derivative P3 exerts its effects in the treatment of T2DM through multiple targets and pathways. Among them, TNF, DRD2, STAT3, IL2, FGF2, and FGF1 are identified as core targets involved in its mechanism of action. The binding energy of P3 and FGF1 was the highest. MD simulations of compound P3 and FGF1 suggest that their binding is stable. Therefore, compound P3 may directly bind to the FGF1 protein to exert its pharmacological effects. The key signaling pathways through which compound P3 functions in the treatment of T2DM include neuroactive ligand–receptor interaction, calcium signaling pathway, PI3K-Akt signaling pathway, and pathways in cancer. We compared compound P3 with the hypoglycemic drug acarbose by performing molecular docking, molecular dynamics simulations, and binding free energy calculations with α-glucosidase. The results demonstrated that P3 exhibits a more stable binding interaction and a lower binding energy (−41.237 kcal/mol) with α-glucosidase. In addition, amino acid residues Lys9, Asn 12, Glu13, Tyr32, Leu162, and Lys280 may play an important role in the catalytic process due to their strong interaction with product P3 in complex. Pharmacokinetic prediction results for compound P3 also indicate that it has excellent biocompatibility. Finally, we constructed the mutant of UGT Bs-YjiC with high site selectivity for the C28 site of betulinic acid. In summary, P3 is a promising lead compound for further development as a T2DM drug.

## Figures and Tables

**Table 1:** Annotation of KEGG pathways with TOP10 enrichment degree and the involved potential targets.

KEGG Pathways	Target Count	Targets
hsa04080: Neuroactive ligand–receptor interaction	15	PTAFR, HTR2B, HTR1B, HTR2C, ADRA1D, HTR2A, F2, ADRA2C, ADRA1A, ADRA2B, ADRA2A, GLRA1, DRD1, DRD2, DRD3
hsa04020: Calcium signaling pathway	10	PTAFR, HTR2B, KDR, HTR2C, ADRA1D, DRD1, HTR2A, FGF1, ADRA1A, FGF2
hsa04540: Gap junction	5	HTR2B, HTR2C, DRD1, HTR2A, DRD2
hsa04726: Serotonergic synapse	5	CYP2D6, HTR2B, HTR1B, HTR2C, HTR2A
hsa05321: Inflammatory bowel disease	4	STAT3, RORC, TNF, IL2
hsa01521: EGFR tyrosine kinase inhibitor resistance	4	STAT3, KDR, FGF2, BCL2L1
hsa04022: cGMP-PKG signaling pathway	5	ADRA1D, ADRA2C, ADRA1A, ADRA2B, ADRA2A
hsa04659: Th17 cell differentiation	4	HSP90AA1, STAT3, RORC, IL2
hsa04151: PI3K-Akt signaling pathway	6	HSP90AA1, KDR, FGF1, FGF2, IL2, BCL2L1
hsa05200: Pathways in cancer	7	HSP90AA1, STAT3, F2, FGF1, FGF2, IL2, BCL2L1
hsa05162: Measles	4	STAT3, TLR9, IL2, BCL2L1
hsa05205: Proteoglycans in cancer	4	STAT3, KDR, TNF, FGF2
hsa04015: Rap1 signaling pathway	4	KDR, DRD2, FGF1, FGF2
hsa05417: Lipid and atherosclerosis	4	HSP90AA1, STAT3, TNF, BCL2L1
hsa04750: Inflammatory mediator regulation of TRP channels	3	HTR2B, HTR2C, HTR2A
hsa05171: Coronavirus disease—COVID-19	4	STAT3, F2, TNF, IL2
hsa04014: Ras signaling pathway	4	KDR, FGF1, FGF2, BCL2L1
hsa05142: Chagas disease	3	TLR9, TNF, IL2

**Table 2:** ADMET profiling of compounds.

Compound	P3
Human Intestinal Absorption	0.37
Oral Bioavailability	0.46
Aqueous Solubility	−6.62 log mol/L
Lipophilicity	2.17 log-ratio
Hydration Free Energy	−7.72 kcal/mol
Cell Effective Permeability	−6.24 cm/s
PAMPA Permeability	0.33
P-glycoprotein Inhibition	0.43
Blood–Brain Barrier Penetration	0.23
Plasma Protein Binding Rate	96.92%
Volume of Distribution at Steady State	−7.8 L/kg
CYP1A2 Inhibition	0.00124
CYP2C19 Inhibition	0.01
CYP2C9 Substrate	0.02
CYP2C9 Inhibition	0.01
CYP2D6 Substrate	0.02
CYP2D6 Inhibition	0.01
CYP3A4 Substrate	0.62
CYP3A4 Inhibition	0.01
Half Life	62.98 h
hERG Blocking	0.44
Clinical Toxicity	0.09
Mutagenicity	0.1
Drug Induced Liver Injury	0.2
Carcinogenicity	0.01
Acute Toxicity LD50	3.39 log(1/(mol/kg))
Skin Reaction	0.31
Androgen Receptor (Full Length)	0.07
Androgen Receptor (Ligand Binding Domain)	0.04
Aryl Hydrocarbon Receptor	0.0047
Aromatase	0.12
Estrogen Receptor (Full Length)	0.22
Estrogen Receptor (Ligand Binding Domain)	0.08
Peroxisome Proliferator-Activated Receptor Gamma	0.17
Nuclear Factor (Erythroid-Derived 2)-Like 2/Antioxidant Responsive Element	0.37
ATPase Family AAA Domain-Containing Protein 5 (ATAD5)	0.03
Heat Shock Factor Response Element	0.05
Mitochondrial Membrane Potential	0.15
Tumor Protein p53	0.13

## Data Availability

Data are contained within the article or Supplementary Materials.

## Supplementary Materials

The following supporting information can be downloaded at: https://www.mdpi.com/article/10.3390/molecules29040878/s1, Figure S1: ^1^H NMR spectrum of P3 in methanol-*d*4 (600 MHz); Figure S2: ^13^C NMR spectrum of P3 in methanol-*d*4 (600 MHz); Figure S3: HSQC spectrum of P3 in methanol-*d*4 (600 MHz); Figure S4: HMBC spectrum of P3 in methanol-*d*4 (600 MHz); Figure S5: COSY spectrum of P3 in methanol-*d*4 (600 MHz); Figure S6: ^1^H NMR spectrum of P4 in methanol-*d*4 (600 MHz); Figure S7: ^13^C NMR spectrum of P4 in methanol-*d*4 (600 MHz); Figure S8: HSQC spectrum of P4 in methanol-*d*4 (600 MHz); Figure S9: HMBC spectrum of P4 in methanol-*d*4 (600 MHz); Figure S10: COSY spectrum of P4 in methanol-*d*4 (600 MHz); Figure S11: Wynn diagram of compound targets and II diabetes targets; Figure S12: Molecular docking of P3 with FGF1; Figure S13: Molecular dynamics simulation of P3 with FGF1; Figure S14: SDS-PAGE diagram of mutant M315F; Figure S15: The mutant M315F activity was analyzed by HPLC. Table S1: ^1^H- and ^13^C-NMR (600 MHz) spectral data for product P3 in methanol-*d*4; Table S2: ^1^H- and ^13^C-NMR (600 MHz) spectral data for product P4 in methanol-*d*4; Table S3: Gene names, degree value, betweenness centrality (BC) and closeness centrality (CC) of key targets; Table S4: Mutant primers.
