# Supplementary material for: A New Potent Inhibitor against α-Glucosidase Based on an In Vitro Enzymatic Synthesis Approach
Source: Molecules. 2024 Feb 16;29(4):878. doi: 10.3390/molecules29040878 (PMC10893485; doi:10.3390/molecules29040878)
Supplement: Supplementary file 1 [file molecules-29-00878-s001.zip › molecules-2857057-supplementary.pdf]

Supplementary Materials

A new potent inhibitors against α-glucosidase based on *in vitro* enzymatic synthesis approach

Huanyu Zhang [a,b #], Xiance Che [c #], Hongyan Jing [c], Yaowu Su [a,b], Wenqi Yang [a,b], Rubing Wang [a,b], Guoqi Zhang [a,b], Jie Meng [a,b], Wei Yuan [a,b], Juan Wang [a,b*], Wenyuan Gao [a,b*]

[a] School of Pharmaceutical Science and Technology, Tianjin University, Tianjin 300072, China;

[b] Key Laboratory of Systems Bioengineering, Ministry of Education, Tianjin University, Tianjin 300072, China;

[c] School of Traditional Chinese Materia Medica, Tianjin University of Traditional Chinese Medicine, Tianjin 301600, China;

Prof. Dr. Wenyuan Gao and Associate Prof. Dr. Juan Wang

School of Pharmaceutical Science and Technology, Tianjin University, Tianjin 300072, P.R. China, Tel: +86 22 87401895; Fax: +86 22 87401895;

Email: pharmgao@tju.edu.cn (Wenyuan Gao, Dr. Gao is fully responsible for the distribution of all materials associated with this article), drwangjuan@tju.edu.cn (Juan Wang)

[#] These authors contributed equally to this work.

1. Supplementary Figures 3
Figure S1. ^{1}H NMR spectrum of P3 in methanol–*d*4 (600 MHz). 3
Figure S2. ^{13}C NMR spectrum of P3 in methanol–*d*4 (600 MHz). 3
Figure S3. HSQC spectrum of P3 in methanol–*d*4 (600 MHz). 4
Figure S4. HMBC spectrum of P3 in methanol–*d*4 (600 MHz). 4
Figure S5. COSY spectrum of P3 in methanol–*d*4 (600 MHz). 5
Figure S6. ^{1}H NMR spectrum of P4 in methanol–*d*4 (600 MHz). 5
Figure S7. ^{13}C NMR spectrum of P4 in methanol–*d*4 (600 MHz). 6
Figure S8. HSQC spectrum of P4 in methanol–*d*4 (600 MHz). 6
Figure S9. HMBC spectrum of P4 in methanol–*d*4 (600 MHz). 7
Figure S10. COSY spectrum of P4 in methanol–*d*4 (600 MHz). 7
Figure S11. Wynn diagram of compound targets and II diabetes targets. 8
Figure S12. Molecular docking of P3 with FGF1. 8
Figure S13. Molecular dynamics simulation of P3 with FGF1. 9
Figure S14. SDS-PAGE diagram of mutant M315F. 9
Figure S15. The mutant M315F activity was analyzed by HPLC. 10
2. Supplementary Tabes 10
Table S1. ^{1}H- and ^{13}C-NMR (600 MHz) spectral data for product P3 in methanol-*d*4 10
Table S2. ^{1}H- and ^{13}C-NMR (600 MHz) spectral data for product P4 in methanol-*d*4 11
Table S3. Gene names, degree value, betweenness centrality (BC) and closeness centrality (CC) of key targets 12
Table S4. Mutant primers 13

1. Supplementary Figures

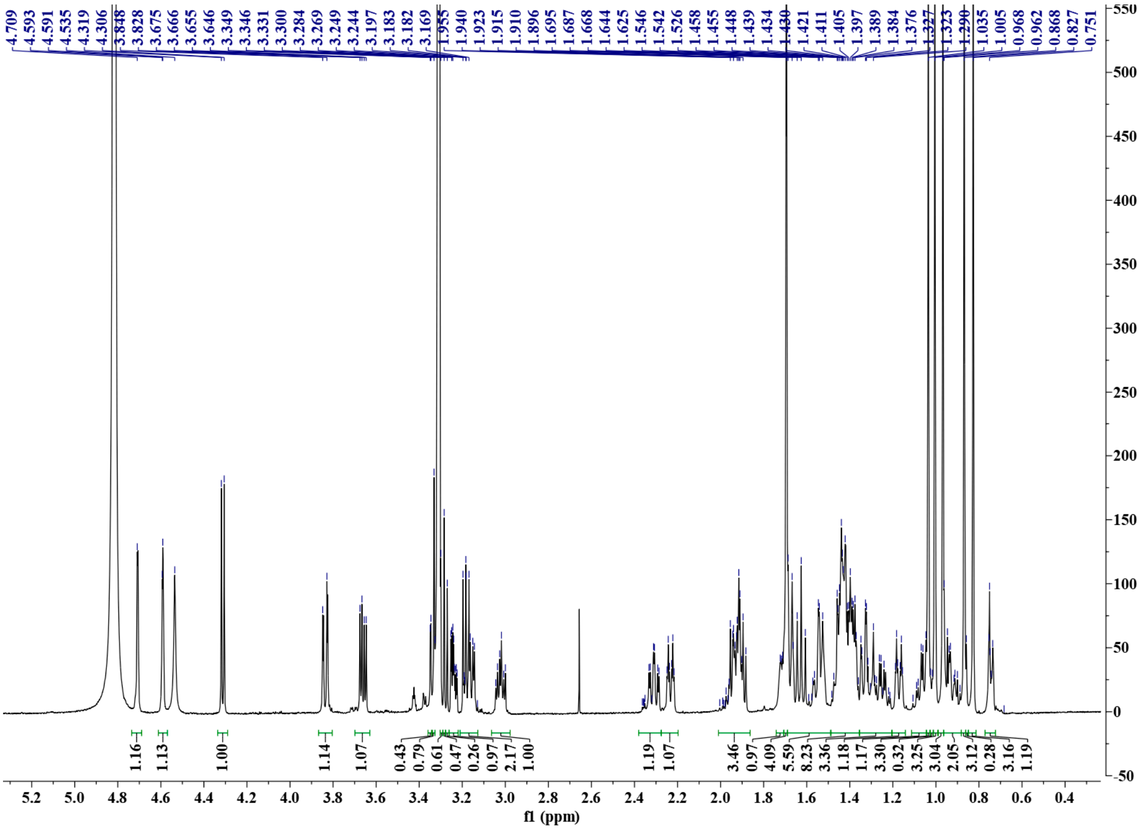

Figure S1. ^{1}H NMR spectrum of P3 in methanol–*d*4 (600 MHz).

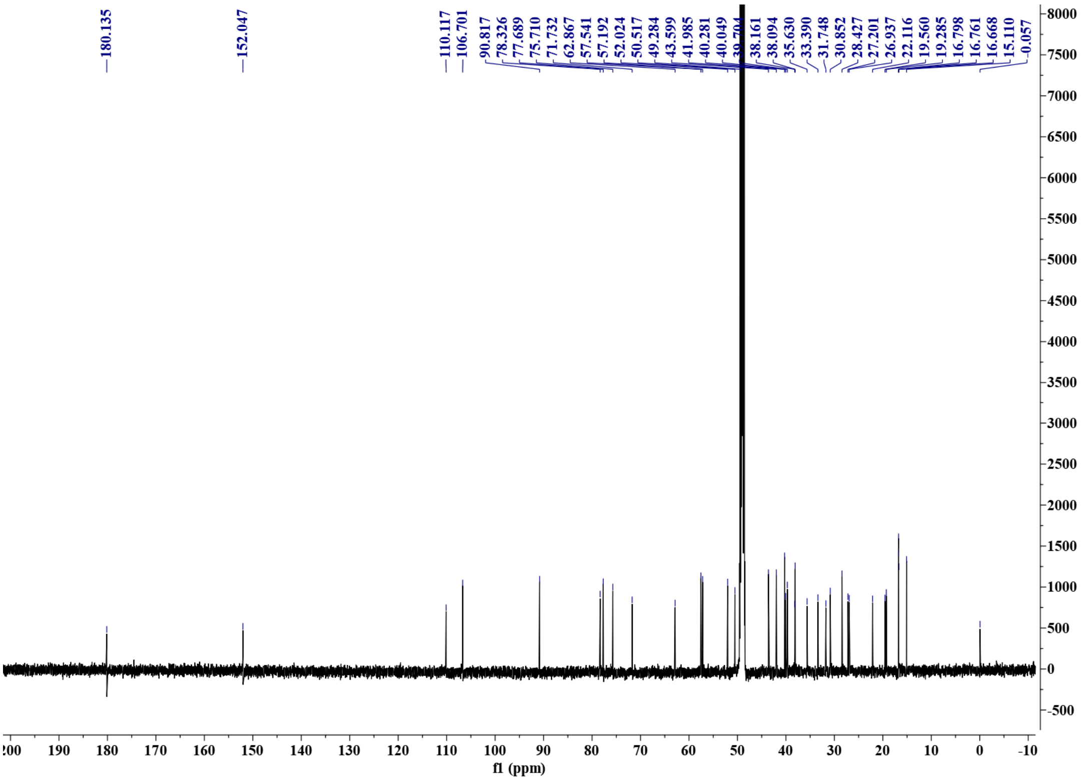

Figure S2. ^{13}C NMR spectrum of P3 in methanol–*d*4 (600 MHz).

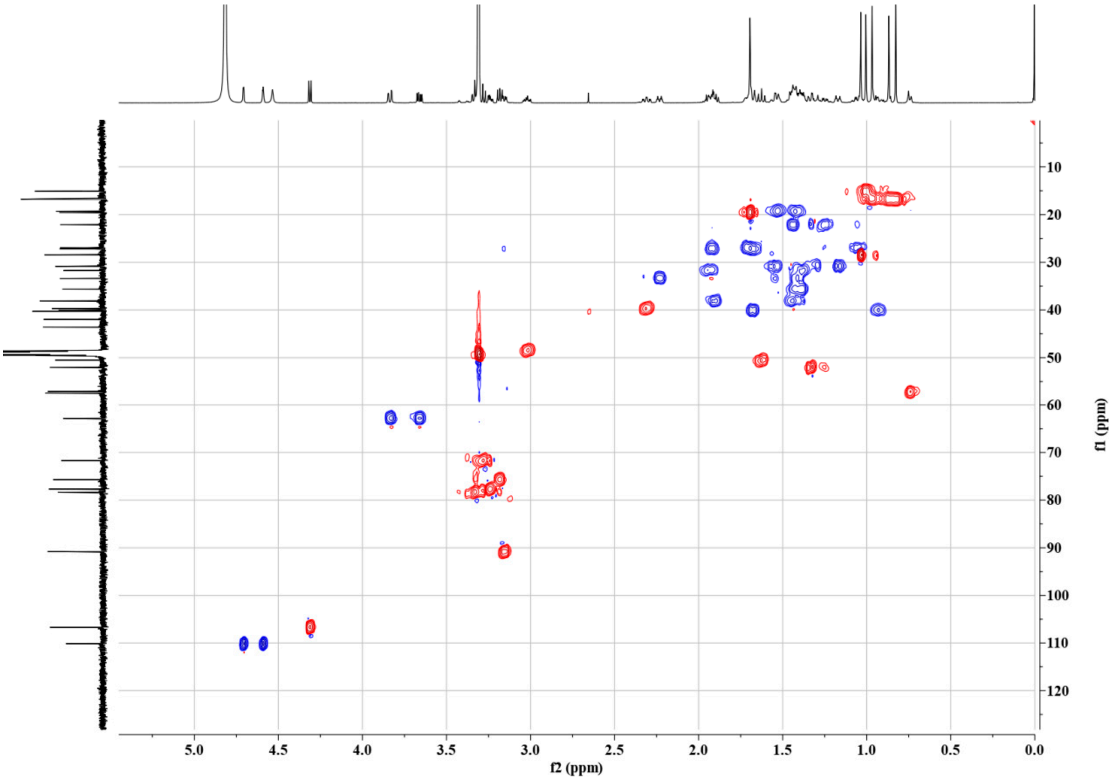

Figure S3. HSQC spectrum of P3 in methanol–*d*4 (600 MHz).

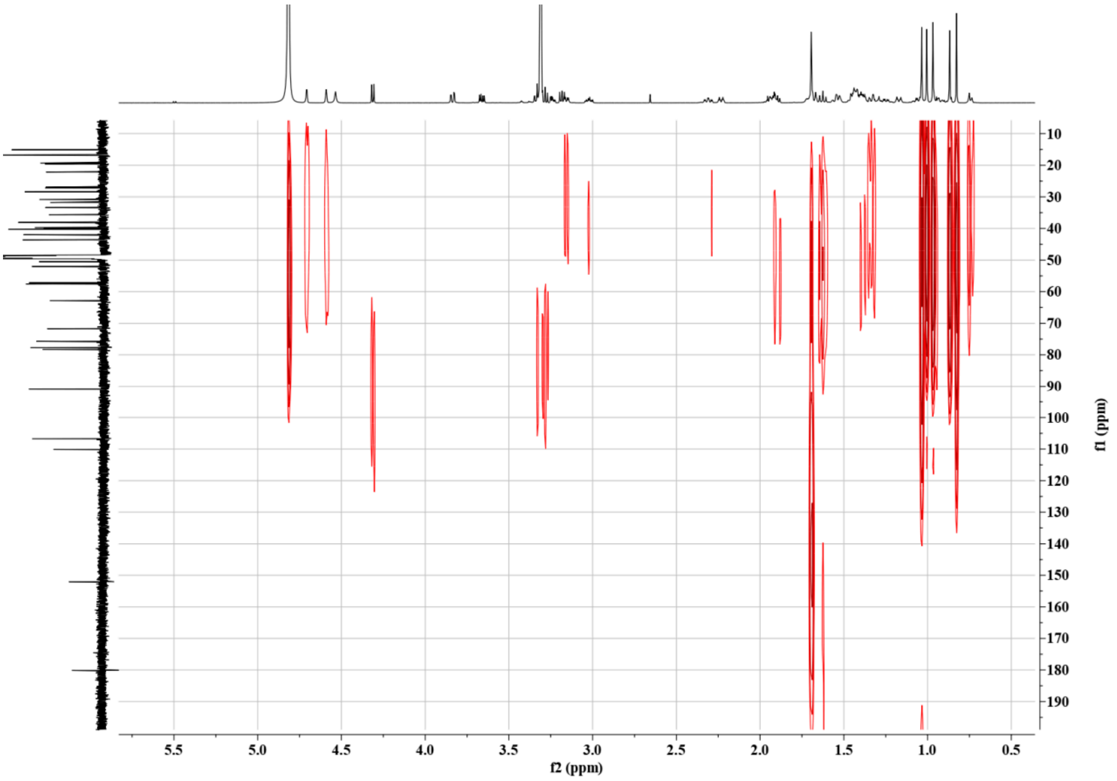

Figure S4. HMBC spectrum of P3 in methanol–*d*4 (600 MHz).

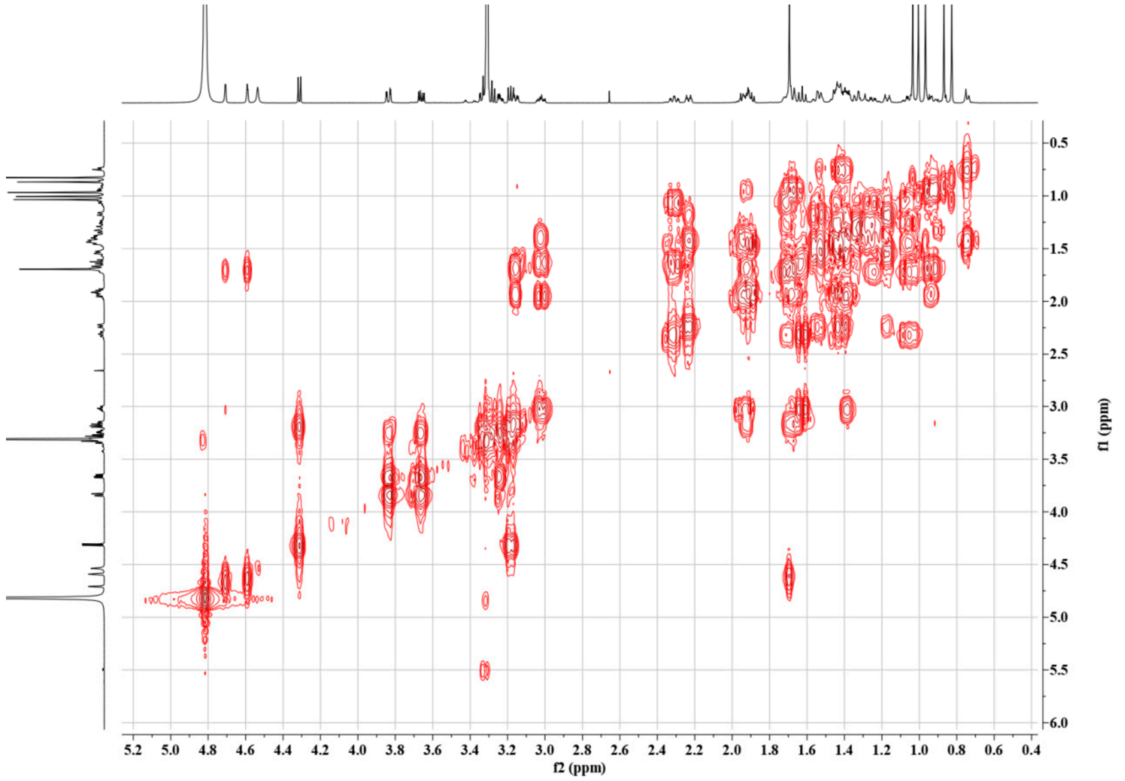

Figure S5. COSY spectrum of P3 in methanol–*d*4 (600 MHz).

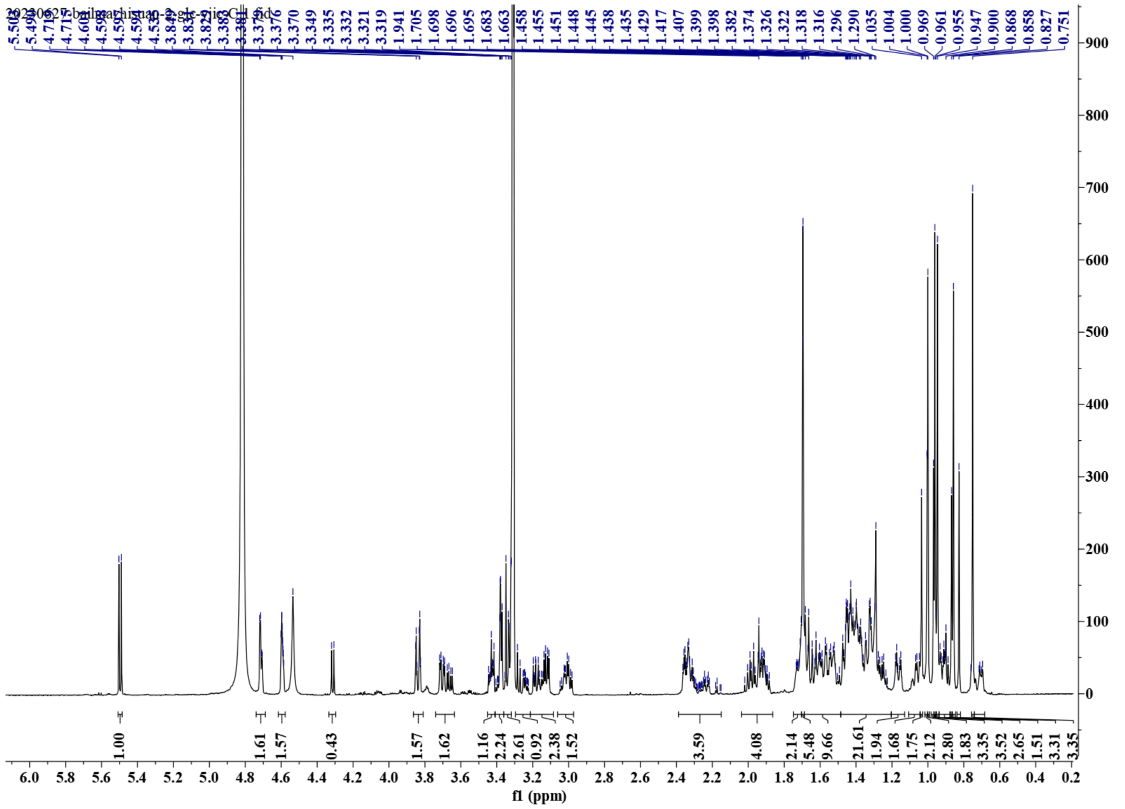

Figure S6. ^{1}H NMR spectrum of P4 in methanol–*d*4 (600 MHz).

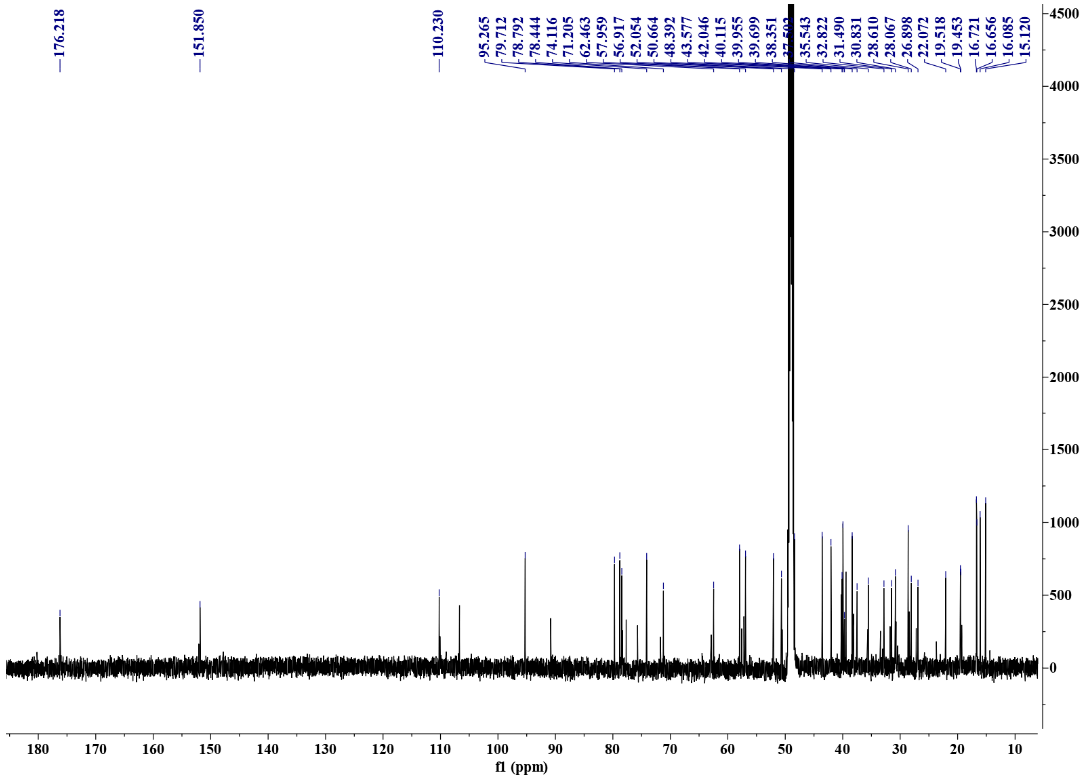

Figure S7. ^{13}C NMR spectrum of P4 in methanol–*d*4 (600 MHz).

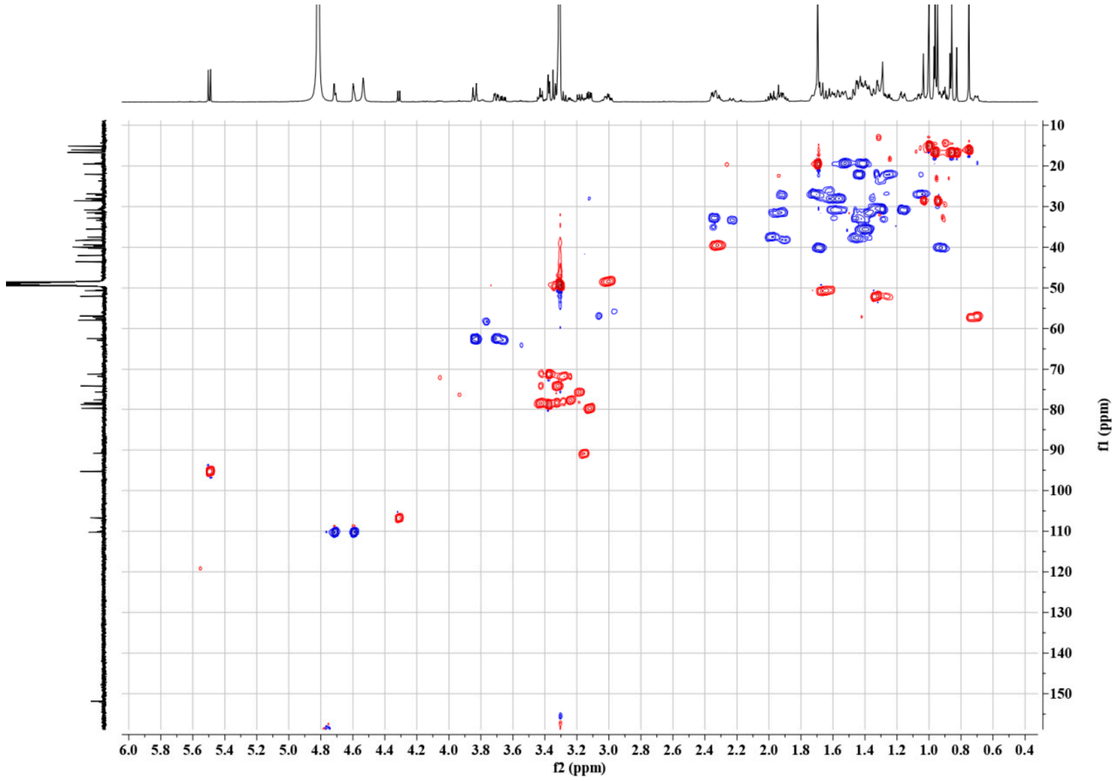

Figure S8. HSQC spectrum of P4 in methanol–*d*4 (600 MHz).

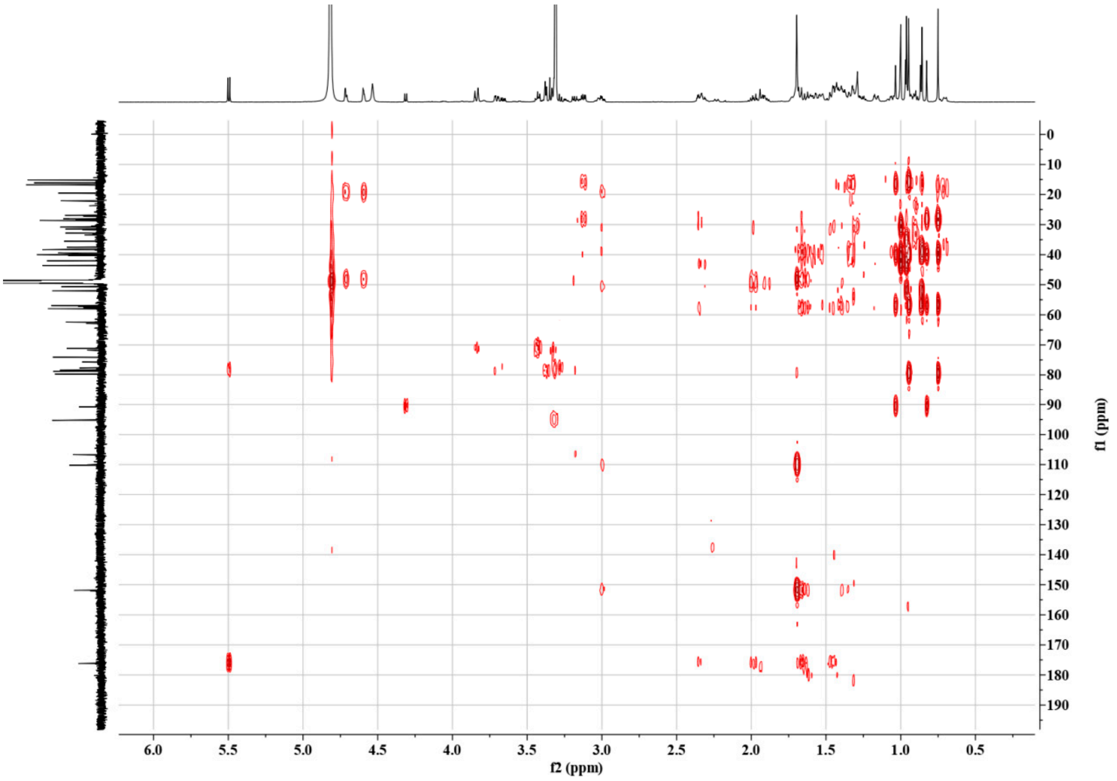

Figure S9. HMBC spectrum of P4 in methanol–*d*4 (600 MHz).

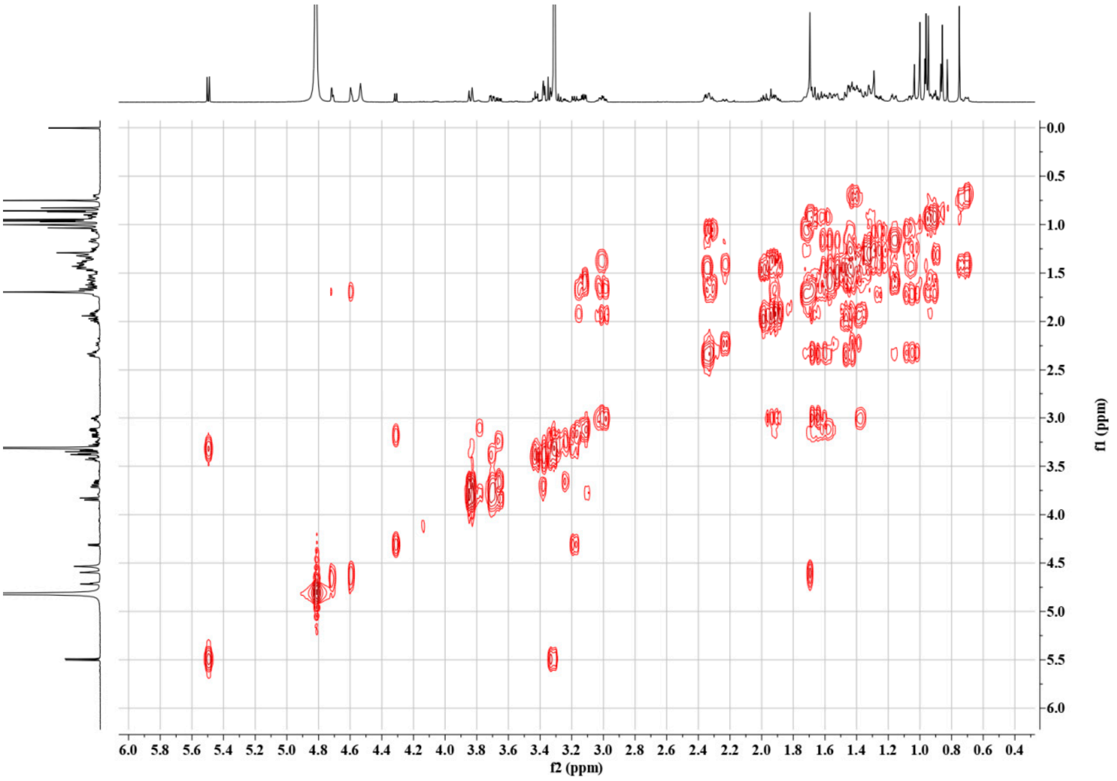

Figure S10. COSY spectrum of P4 in methanol–*d*4 (600 MHz).

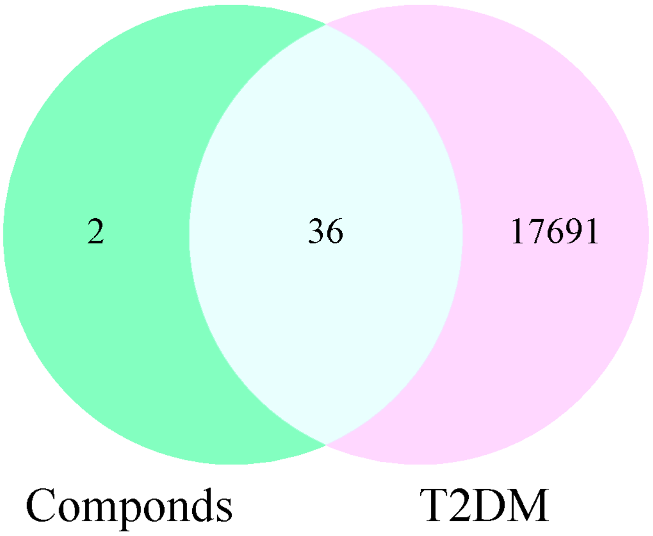

Figure S11. Wynn diagram of compound targets and II diabetes targets.

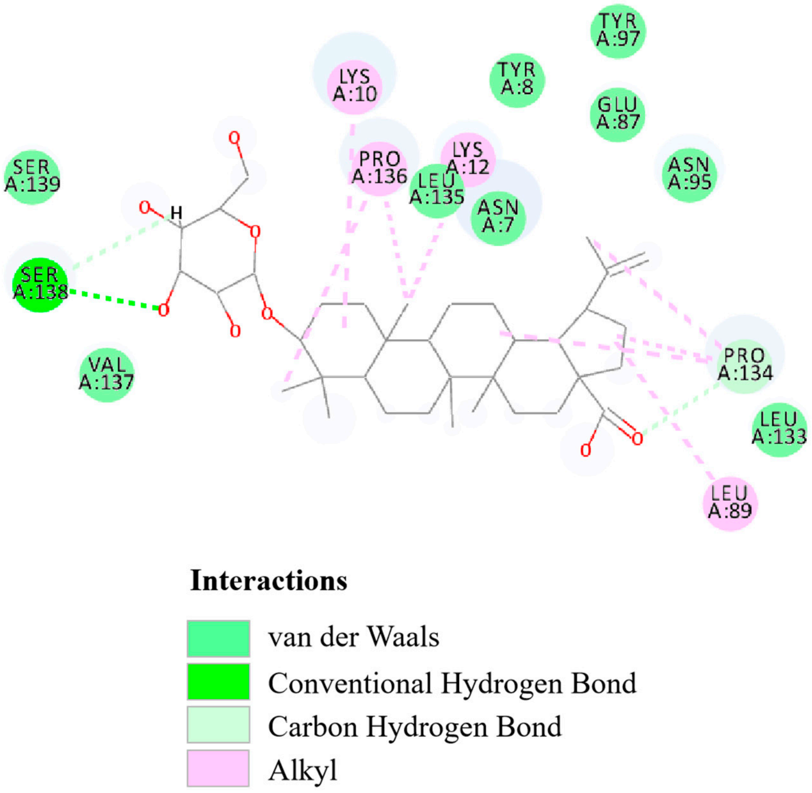

Figure S12. Molecular docking of P3 with FGF1.

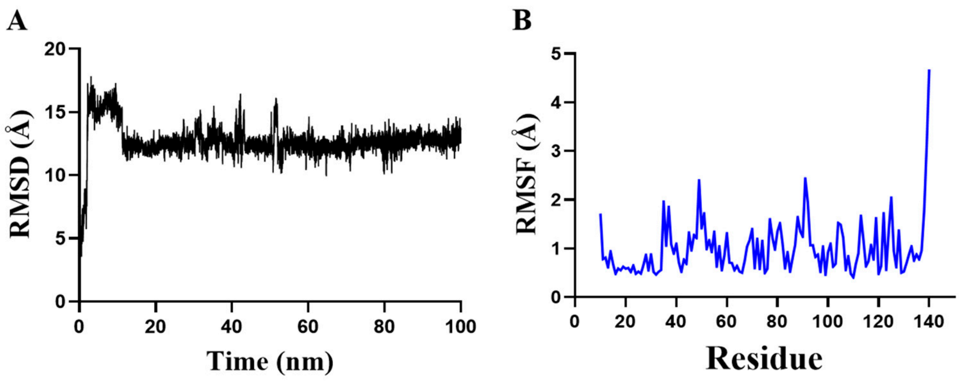

Figure S13. Molecular dynamics simulation of P3 with FGF1.

(A) Time dependence of root mean square deviation (RMSD) of the complex P3/FGF1; (D) Decomposition of binding free energy of residues for FGF1.

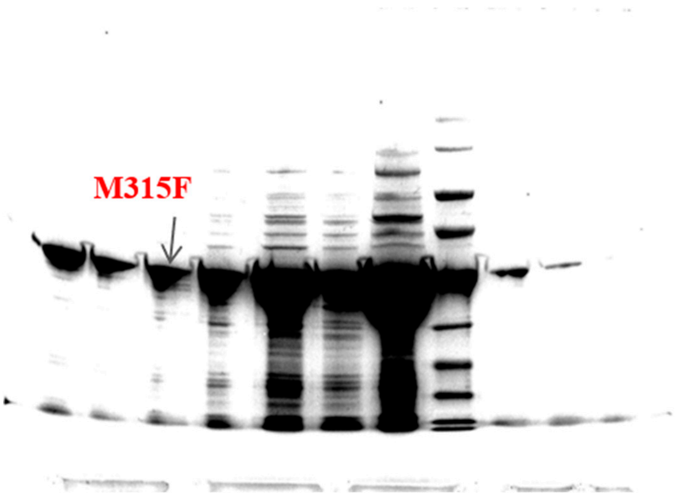

Figure S14. SDS-PAGE diagram of mutant M315F.

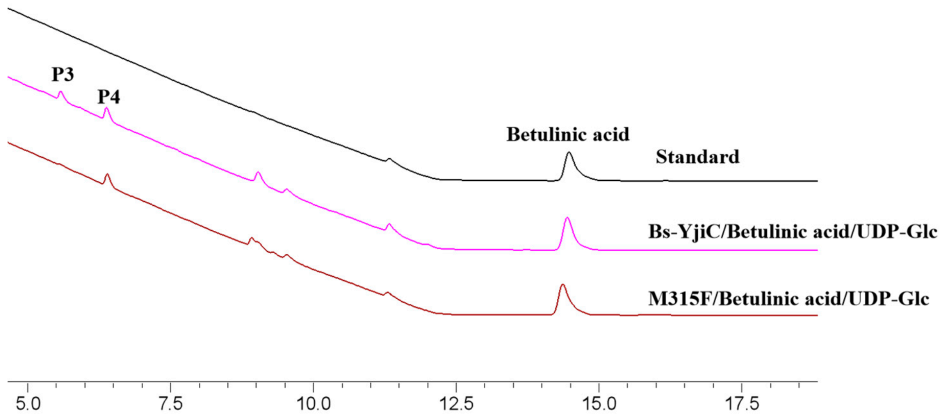

Figure S15. The mutant M315F activity was analyzed by HPLC.

2. Supplementary Tabes

Table S1. ^{1}H- and ^{13}C-NMR (600 MHz) spectral data for product P3 in methanol-*d*4

C	δC (ppm)	δH (ppm)
1	40.0	CH2, 0.93 (m), 1.68 (m)
2	27.2	CH2, 1.68 (m), 1.92 (m)
3	90.8	CH, 3.15 (m)
4	40.3	
5	57.2	CH, 0.74 (m)
6	19.3	CH2, 1.42 (m), 1.53 (m)
7	33.4	CH2, 1.41 (m), 2.23 (m)
8	42.0	
9	52.0	CH, 1.33 (m)
10	38.2	
11	22.1	CH2, 1.25 (m), 1.44 (m)
12	26.9	CH2, 1.04 (m), 1.71 (m)
13	39.7	CH, 2.31 (m)
14	43.6	
15	30.8	CH2, 1.17 (m),1.55 (m)
16	35.6	CH2, 1.39 (m), 1.44 (m)
17	57.5	
18	50.5	CH, 1.63 (m)
19	48.5	CH, 3.02 (m)
20	31.7	CH2, 1.37 (m), 1.91 (m)
21	38.1	CH2, 1.44 (m), 1.91 (m)
22	152.1	

23	28.4	CH3, 1.04 (s)
24	16.7	CH3, 0.82 (s)
25	16.8	CH3, 0.97 (s)
26	16.8	CH3, 0.87 (s)
27	15.1	CH3, 1.00 (s)
28	180.1	
29	110.1	CH, 4.59 (m); CH,4.71 (m)
30	19.5	CH3, 1.69 (s)
3-O-Glc-1′	106.7	CH, 4.31 (*d*, J=7.8 Hz)
3-O-Glc-2′	75.7	CH, 3.19 (m)
3-O-Glc-3′	78.3	CH, 3.33 (m)
3-O-Glc-4′	71.7	CH, 3.28 (m)
3-O-Glc-5′	77.7	CH, 3.24 (m)
3-O-Glc-6′	62.9	CH2, 3.66 (m), 3.83 (m)

Table S2. ^{1}H- and ^{13}C-NMR (600 MHz) spectral data for product P4 in methanol-*d*4

C	δC (ppm)	δH (ppm)
1	40.0	CH2, 0.92 (m), 1.68 (m)
2	28.1	CH2, 1.60 (m), 134 (m)
3	79.7	CH, 3.12 (m)
4	39.7	
5	56.9	CH, 0.70 (m)
6	19.5	CH2, 1.41 (m), 1.52 (m)
7	35.6	CH2, 1.40 (m), 2.35 (m)
8	42.1	
9	52.0	CH, 1.33 (m)
10	38.4	
11	22.1	CH2, 1.23 (m), 1.44 (m)
12	27.0	CH2, 1.04(m), 1.70 (m)
13	40.1	CH, 1.68 (m)
14	43.6	
15	31.5	CH2, 1.37 (m),1.94 (m)
16	32.8	CH2, 1.45 (m), 2.35 (m)
17	58.0	
18	50.7	CH, 1.65 (m)
19	48.4	CH, 3.00 (m)
20	30.8	CH2, 1.12 (m),1.59 (m)
21	37.5	CH2, 1.45 (m),1.98 (m)
22	151.8	

23	28.6	CH3, 0.95 (s)
24	16.1	CH3, 0.75 (s)
25	16.7	CH3, 0.86 (s)
26	16.7	CH3, 0.96 (s)
27	15.2	CH3, 1.00 (s)
28	176.9	
29	110.2	CH, 4.60 (m), CH,4.72 (m)
30	19.5	CH3, 1.70 (s)
28-O-Glc-1′	95.3	CH, 5.50 (*d*, J=6 Hz)
28-O-Glc-2′	74.1	CH, 3.32 (m)
28-O-Glc-3′	78.4	CH, 3.43 (m)
28-O-Glc-4′	71.2	CH, 3.34 (m)
28-O-Glc-5′	78.8	CH, 3.38 (m)
28-O-Glc-6′	62.5	CH2, 3.71 (m), 3.83 (m)

Table S3. Gene names, degree value, betweenness centrality (BC) and closeness centrality (CC) of key targets

Gene name	Degree	Closeness centrality	Clustering coefficient
TNF	14	0.46875000	0.34065934
STAT3	11	0.54545455	0.49090909
DRD2	11	0.56603774	0.29090909
IL2	10	0.42857143	0.60000000
FGF2	9	0.42253521	0.75000000
FGF1	8	0.41666667	0.78571429
KDR	8	0.41666667	0.82142857
CYP2D6	8	0.46875000	0.28571429
HSP90AA1	7	0.41095890	0.95238095
HTR2A	7	0.44117647	0.52380952
DRD1	7	0.40540541	0.47619048
ADRA1D	7	0.41666667	0.61904762
HTR2C	7	0.44117647	0.47619048
BCL2L1	6	0.40540541	1.00000000
ADRA2B	6	0.40000000	0.73333333
ADRA2A	6	0.34883721	0.60000000
ADRA1A	6	0.34883721	0.60000000
TLR9	5	0.40000000	1.00000000
HTR1B	5	0.42857143	0.80000000
HTR2B	4	0.32608696	0.50000000
COL18A1	4	0.33333333	1.00000000
ADRA2C	4	0.38961039	0.83333333
RORC	3	0.38961039	1.00000000

DRD3	3	0.40000000	0.66666667
HSD11B1	3	0.45454545	0.00000000
CYP27B1	3	0.45454545	0.00000000
ALDH2	3	0.40000000	0.66666667
PTAFR	2	0.38461538	1.00000000
F2	2	0.32608696	1.00000000
HSD11B2	2	0.32258065	0.00000000
GLRA1	1	0.24793388	0.00000000

Table S4. Mutant primers

Primers	Sequence (5’to 3’)
M315F-F	CGCTGGTGGTGATTCCGCAGTTTTATGAACAAGAACTGACCGC
M315F-R	GCGGTCAGTTCTTGTTCATAAAACTGCGGAATCACCACCAGCG
